# Indexing Multivariate Mobile Data through Spatio-Temporal Event Detection and Clustering

**DOI:** 10.3390/s19030448

**Published:** 2019-01-22

**Authors:** Reza Rawassizadeh, Chelsea Dobbins, Mohammad Akbari, Michael Pazzani

**Affiliations:** 1 Department of Computer Science, University of Rochester, Rochester, NY 14620, USA; 2 School of Information Technology and Electrical Engineering, University of Queensland, Brisbane 4067, Australia; c.m.dobbins@uq.edu.au; 3 Department of Computer Science, University College London, London WC1E 6BT, UK; m.akbari@ucl.ac.uk; 4 Department of Computer Science, University of California, Riverside, CA 92507, USA; michael.pazzani@ucr.edu

**Keywords:** spatio-temporal, clustering, event detection, mobile sensing: contrast behavior mining, human behavior

## Abstract

Mobile and wearable devices are capable of quantifying user behaviors based on their contextual sensor data. However, few indexing and annotation mechanisms are available, due to difficulties inherent in raw multivariate data types and the relative sparsity of sensor data. These issues have slowed the development of higher level human-centric searching and querying mechanisms. Here, we propose a pipeline of three algorithms. First, we introduce a spatio-temporal event detection algorithm. Then, we introduce a clustering algorithm based on mobile contextual data. Our spatio-temporal clustering approach can be used as an annotation on raw sensor data. It improves information retrieval by reducing the search space and is based on searching only the related clusters. To further improve behavior quantification, the third algorithm identifies contrasting events *within* a cluster content. Two large real-world smartphone datasets have been used to evaluate our algorithms and demonstrate the utility and resource efficiency of our approach to *search*.

## 1. Introduction

The proliferation of mobile and wearable devices offers researchers opportunities to detect, identify, and classify human behavior. New computational paradigms, in combination with wearable sensors have powered the “quantified self” and “mobile health” movements, in addition to the renewal of interest in underutilized paradigms, such as “lifelogging” and “personal informatics” among other terms for self-tracking to improve personal performance. All of these systems benefit from sensor data that have been collected by mobile and wearable devices from the user’s environment and behavior. These data are *multivariate* (e.g., accelerometer and GPS), typically *sparse* and *time-stamped* [1].

However, despite the richness of this data, there remains a lack of appropriate searching and information retrieval mechanisms that are able to filter sensor data within the resource limitations of small mobile devices. Therefore, data analysis should be done on a remote host, such as on the cloud or cloudlet [2]. However, this will raise network response time and privacy related issues [3].

We believe virtual assistance devices or applications with conversational agents could work in synergy with human memory, enabling users to recall previous life events through lifelogging. For example, a user can ask her virtual assistant *“How many times did I visit the gym last month?”* or *“How long did I spend in traffic during my daily commute?”*. In spite of the obvious utility of such a system, such search and querying mechanisms for mobile health applications (e.g., Google Fit [4], Samsung Health [5] and FitBit [6]) on personal assistants (e.g.,  SiRi [7] and Cortona [8]) do not yet exist. Existing mobile health applications continuously collect data, but they only provide temporal browsing and graph based visualizations. Graph illiteracy is a major challenge among individuals, even in developed countries [9], that has affected the usability of mobile health applications [10].

Any requirement for manual intervention in these systems is a barrier to adoption [11]. Therefore, frequently annotating the data manually is not useful. However, manual annotation is inevitable and we cannot completely remove it. On the other hand, little work has addressed index creation from *multivariate temporal sensor data*. An annotation/indexing mechanism can facilitate higher-level searching and querying of datasets composed of temporal sensor data.

In response to these challenges, this paper is aimed at *spatio-temporal indexing* of multivariate temporal data to reduce the search space for facilitating human-centric searching of queries.

**Figure 1:**
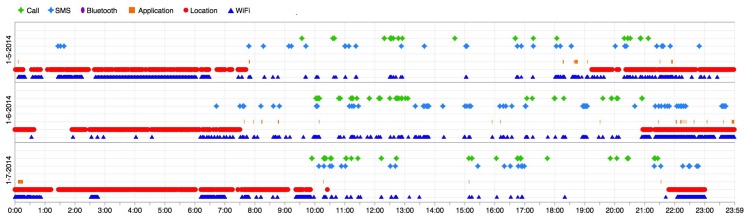
UbiqLog life log visualization of three days of data for a single user (best viewed in color).

Our approach is toward enabling mobile devices to search their collected data in a reasonable time with less resource utilization. In particular, our contribution is a pipeline of three algorithms that improves search execution time and resource-efficiency, as follows:

- We describe a **spatial event detection** algorithm to detect daily life events from raw data (mobile sensor data). Daily life events are typically grounded in specific times and locations, and this spatio-temporality can be extracted from sensor data. Converting daily activities into discrete spatial events is our first step toward annotating and indexing the raw data. Since location data from mobile devices are sparse and not always available, our algorithm should be able to cope with uncertainty and sparsity. For instance, Figure 1 shows a visualization of three days of data from a user. It shows that location data (•) and WiFi data (▲), which could be used for location estimation, are not always available.
- Given that human mobility behavior is known to be predictable, at least in the aggregate [12], our second contribution is an **unsupervised spatio-temporal clustering** mechanism that identifies similar daily life-events and annotates them based on their correlation with *location changes* and *times*. In other words, life events during a routine behavior, e.g., commuting to a work at a specific time of the day, or going to the movies on weekends, will tend to map to the same cluster.
  This spatio-temporal clustering provides a higher level of annotation (index), and in turn reduces the search space.
- Our third contribution is exploiting the content of each individual cluster to allow us to identify **contrasting events inside a cluster**. The identification of contrasting events (behaviors) is a major step toward the enrichment of sensor data *inside* a cluster, and thus refining the described spatio-temporal indexes. For example, consider a user who visits a coffee shop for two purposes, either to chat with friends or to work. Since both chatting and working take place in the same location, and, at the same time, spatio-temporal event detection alone may not suffice to distinguish between these two distinct user behaviors. However, data from the mobile or wearable device microphone can differentiate between working and chatting (at the same location/time). Therefore, a contrast-set detection [13] method is better positioned to delve deeper into the content of our spatio-temporal clusters. Furthermore, first searching clusters with fewer contrasting events could improve search execution time as well.

To concretely ground these concepts, consider two application scenarios that would benefit from using our algorithms:

(i) User 1 goes to the gym on a regular basis, and maintains her diet. Nevertheless, she starts gaining weight. Using the contrast event detection algorithm, she realizes that she recently began spending less time on cardio training in favor of weight training, which is a prime suspect for her weight gain.
(ii) User 2 has a flexible working schedule. Through the spatio-temporal event detection algorithm, he can estimate how much time he spends commuting to work on average, and then find out the best time/day to commute.

These two examples exhibit the utility of using the spatio-temporal annotation (indexing) within higher-level applications. In other words, our algorithms facilitates searching through *reducing the search space* based on *clustering* and analyzing clusters based on their *contrasting events*. Furthermore, prioritizing the search based on clusters with a lower number of contrasting events could slightly improve the search execution time. There are promising approaches for analyzing mobile data to extract behavioral patterns [1,14,15]. However, based on our knowledge, this is the first work that relies on the *spatio-temporality* of the mobile data for annotating other sensor data, clustering them and ordering the clusters based on their contrasting events. In other words, this is the first work to employ such a facility toward enabling on-device [3] search operations for end users.

Note that, except event detection, which does not have any new parameters, each of our algorithms has only one parameter to configure (in total two parameters, one for spatial clustering and one for contrast behavior detection) and, in the evaluation section, we report parameter sensitivity analysis in detail. Parameters for the event detection are either constant or they have been chosen based on optimal values from previous works. Each algorithm could be used separately as well. For instance, a user can choose another event detection method, not using contrast behavior detection and only use the temporal clustering algorithm.

Implementing the end-user application which hosts this search facility is not in the scope of this paper. The end-user query parsing is the task of the application that hosts our methods. Moreover, our algorithms are not able to completely remove the burden of manual location annotation, despite mitigating it significantly. They can be used inside applications (as middleware) that require searching mobile data or converting raw data to higher-level spatio-temporal information, and reducing the need for manual annotation.

## 2. Problem Statements

Daily events in a person’s life (e.g., going to the gym) can be recognized by mobile and wearable devices. Here our focus is on *spatio-temporal* daily life events, or in other words activities of daily life. As such, this work does not directly support higher-level and longer term life events, such as getting married or attending graduate school. Moreover, life events contain nested events, which are not covered in this work, e.g., being at work may be associated with nested events such as drinking coffee, moving to different offices, etc. Our work can distinguish *discrete* daily life activities, based on *location changes*, such as driving to work or going to a restaurant.

Notwithstanding these limitations, we believe this work is among the first toward quantifying spatio-temporal dynamics from sparse multivariate temporal data from smartphones.

### 2.1. Spatial Event Detection

Daily life *events* occur in *a specific location* at *a specific time*, and can be understood as a set of *actions* from the device a user is carrying. Each action, *a*, can be modeled within a 4-tuple arrangement: a=<S,D,T,L>. *S* denotes the context sensor name (or attribute name), *D* is sensor data (or value of the attribute), *T* is the timestamp of the action, and *L* is location of the action. Geographical coordinates in most real-world cases does not exist. Therefore, we focus only on location state and accurate geographical coordinates, i.e., *L*. Each user life event can be formalized within a specific time *T* and specific location *L*. Previous works [14,16] have proposed using spatial changes as a primitive. However, a single location can be associated with disparate actions, i.e., home, work, etc. Thus, we need to consider the finer granularity of actions used to model an event. In other words, an event *e* is composed of a finite set of actions: $e=\{a_{1},a_{2},a_{3},\ldots ,a_{n}\}$ and all these actions have the same location state (specific constant location). If the location has changed, it is the end of the current event and the beginning of a new event. *L* is the constant in the following definition of an event: $e=\{<S_{1},D_{1},T_{1},L>,<S_{2},D_{2},T_{2},L>,\ldots ,<S_{n},D_{n},T_{n},L>\}$. Therefore, the event *e* can be described as:

(1) $$e=\{a_{i}\;:\;l\left(a_{i}\right)\in \left(\Theta \vee \emptyset \right),\cup l\left(a_{i}\right)=l,T_{min}\leq t\left(a_{i}\right)\leq T_{max}\},$$

where l(a) is the location, and t(a) is the time of the action *a*, while each event is bounded by specific temporal borders. By constant location, we do not mean the exact latitude and longitude of a location (For the sake of simplicity, we refer to the movement state, location changes, as ’location state’); we mean a movement state i.e., *moving*, *stationary* and *unknown*. Unknown is used when a location is not available, e.g., in Figure 1 between ∼1:30 p.m. to ∼3:00 p.m. on 1 July 2014, because there is no WiFi and geographical coordination data available. Here, the process of location annotation is manually assigning labels to the identified location state, based on time of the day, which is used in our evaluation section. The first question is to identify l(a), which represents the location. Since it is constant in each event, we use **Θ** to denote the location, either “moving” or “stationary”. Due to pervasive device restrictions, e.g., GPS does not work indoors, the user turns it off, etc., it is not possible to continuously get location, thus l(a) is either null or Θ. In addition, the union of all locations of actions inside an event are a single location state. At this point, Θ is a geographical coordinates object (if it exists). Later, it will be a location state, which we will describe it in this section.

### 2.2. Temporal Clustering

To index events, the second challenge is to identify and cluster similar events that occur (i) at about the same time interval in consecutive days and (ii) in the same location state (moving, stationary or unknown). For instance, events that include “daily commute to work” will be assigned to one cluster, and events that include “attending the gym in the evening” will be assigned to another cluster. Note that this clustering will be done for each user separately and no information will be shared between users.

Routine human behaviors do not usually occur at a fine temporal granularity [1], and there is a temporal uncertainty. For instance, a person may go for a coffee break one day at 4:34 p.m., the next day at 4:31 p.m. and the day after that at 4:20 p.m.. Given this, any model of human behavior should be flexible enough to be invariant to such minor timing differences.

To handle this need for flexibility in our model, we define a *temporal interval*, λ. This interval will be used to mitigate the inevitable time “slope” of the time of event occurrences. In other words, λ will be used for comparison between the start of events (lower bound) and also the end of events (upper bounds). Later, in Section 4, we will describe more about the use of λ. With this notation, we formally define the task of temporal clustering of events as the following:

**Definition** **1.** *Given a set of events C and a temporal interval λ, the objective is to categorize events into k different sub-groups, i.e., clusters, ${\left\{C_{i}\right\}}_{i=1}^{k}$. For each pair of events $e_{x}$, $e_{y}$ in any cluster $C_{i}$ the following constraint holds, where $e_{x}\left(a\right)$ presents an action a inside event $e_{x}$.*

(2) $$\forall \left(e_{x},e_{y}\right)\in C_{i}:\left\{\begin{array}{c} |min(t\left(e_{x}\left(a\right)\right)-min\left(t\left(e_{y}\left(a\right)\right)\right|\leq \lambda ,\\ max(t\left(e_{x}\left(a\right)\right)-max(t\left(e_{y}\left(a\right)\right)\leq \lambda , \end{array}\right.$$

In other words, for each pair of events $e_{x}$ and $e_{y}$, the temporal difference between their lower and upper bounds should not exceed the λ temporal interval. However, there are events that could be routine, but they occur at very different times of the day. Those events cannot be handled by our approach.

### 2.3. Contrasting Events Identification

One can argue that simply clustering based on spatio-temporal properties of activities is too much of a generalization for human behavior. For instance, a user could go to the gym some times for cardio training and sometimes for weight lifting. In these instances, the spatio-temporality of both events are similar, i.e., they stay in the same cluster. However, they are different behaviors. This example shows that we need to have a deeper overview of behaviors.

**Figure 2:**
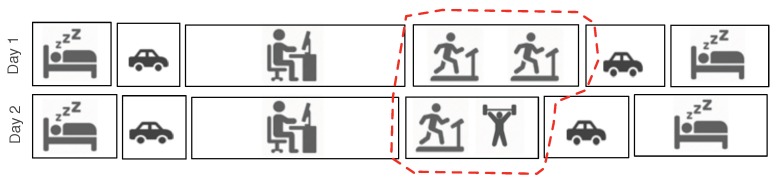
A presentation of constrasting activities bewteen two evetns of the gym cluster (the cluster is marked with red dotted area), i.e., cardio training and weight lifting.

After temporal clustering, we go one step further and compare/contrast the contents of each individual cluster. This is an important step toward augmenting cluster content. Consider the previous example in which a person regularly visits the same place each weekend. Sometimes, she performs weight lifting and sometimes she performs cardio training. Figure 2 is a toy example that visualizes a daily routine behaviors of a user. In this scenario, the spatial and temporal similarity failed to distinguish the contents of her activities inside the gym, and thus we need to examine more closely the activities that are happening “inside” the cluster.

Traditionally, contrast-set mining is used to identify meaningful differences between groups by finding predictors that discriminate between the groups [13]. Inspired by this concept, we define that two events $e_{m}$ and $e_{n}$ inside a cluster are contrasting/dissimilar if they fail to share at least ω number of common actions, where ω denotes the *dissimilarity threshold* controlling the flexibility of the algorithm. For example, in Figure 2, if ω=1 the events in the gym cluster are all considered similar. However, if ω=2, then the two gym events in that cluster are considered to be dissimilar.

Contrasting events can be identified by the following $\Gamma_{c}$ function:

(3) $$\Gamma_{c}\left(e_{m},e_{n}\right)=\left\{\begin{array}{c} 1,\;if\;\sum_{i,j=1}^{i\leq m,j\leq n}e_{m}\left(a_{i}\right)\cap e_{n}\left(a_{j}\right)\geq \omega ,\\ 0,\;otherwise, \end{array}\right.$$

where e(a) function denotes all *actions* inside the event *e*. If the comparison between two events returns fewer than ω actions, then the $\Gamma_{c}$ output is false (0), otherwise this function returns true (1). False output means these events are not significantly different (i.e., not contrasting behaviors). Our experiments mostly show that events inside a cluster have a similar number of actions; therefore, there is no need to use a Jaccard similarity, just relying on the intersection is enough. Going back to the example of User1, if ω number of *actions* (e.g., physical activities that can be measured via an accelerometer) that she performs in her two gym sessions are different, then the two gym events are considered contrasting events, e.g., cardio versus free weights. Therefore, the problem of contrast event detection can be formalized as:

**Problem** **1.** Given a cluster of events $C_{i}$ and a dissimilarity threshold ω, the objective is to identify contrasting events inside the $C_{i}$ cluster by detecting any pair of events $e_{m}$ and $e_{n}$, which holds $\Gamma_{c}\left(e_{m},e_{n}\right)=1$.

This problem formulation exploits the intrinsic multivariety of the data. In particular, our experimental datasets contain data objects from different resources (multivariate), but this model links them together via timestamps and converts different timestamped data as fine-grained units.

## 3. Datasets

Two smartphone datasets have been used for our experiments, UbiqLog [17] and Device Analyzer [18]. We chose these two datasets because in the real-world there are many different makes and models of smartphones, and each device has its own restrictions and specifications for hardware and software. This affects the quantity and quality of the data. As we are demonstrating our algorithms in a real-world setting, we have chosen these two datasets because both have collected data outside a lab setting in the real world.

**UbiqLog** [19]: An open source Android-based life logging tool [20] has been used to create the UbiqLog dataset. It includes 9.78 million records of users’ detected WiFi, Bluetooth, application usage, SMS, call, physical activity (based on Google Play Services) and geographical location. The capacity for location extraction varies based on availability and device status, e.g., GPS, Cell-ID and Google Play Services Location API. Figure 1 shows a visualization of three days of a single user’s data in the UbiqLog dataset. The *x*-axis represents the time of the data, and each sensor has a different color. Table 1 provides details about the UbiqLog dataset records, whilst another report [11] describes more about the data collection experiment. All records are semantically rich and are human readable. Therefore, there is no raw sensor data, such as accelerometer data, in this dataset.

**Device Analyzer** [21]: This is the largest smartphone data-set created and is based on hardware status and device configuration of Android smartphones in which users have installed the Device Analyzer app. It contains raw sensor data for more than 30,000 users. However, our interest is only in the data that is common with the UbiqLog data. This is because UbiqLog is focused on human-centric data that can be collected via smartphones. Device Analyzer is a more hardware-oriented approach and includes detailed information about device status changes. Table 2 shows the number of records for the 35 random Device Analyzer users.

Although the real focus of our algorithms is on user-centric data, to demonstrate the versatility of our approach, we have performed our evaluations on both datasets. We have randomly selected 35 users from Device Analyzer and 35 users from UbiqLog, which means in total we have experimented on 70 users.

## 4. Algorithms

The first step of our approach is to extract events based on the spatio-temporal properties of sensor data, for each user. To identify events from raw data, we introduce a spatial change point detection method. Then, based on both temporal borders and spatial state, we introduce temporal interval based clustering to group similar events together. As it has been described, spatio-temporal quantification alone may not suffice for all application scenarios. Therefore, elements of each cluster are further analyzed to identify dissimilar events. Since privacy issues currently appear insurmountable [22,23], all proposed algorithms are completed separately for each user, and information is not shared among users.

### 4.1. Spatial Change Point Detection

Event identification is based on location state changes. As described, location refers to *moving*, *stationary* or *unknown*. This notion of location is more limited than in other research efforts, which consider geographical locations. However, in the real world, we do not have access to the geographical coordinates 24/7. Therefore, this definition has the advantage of greater availability, which is required in a real-world application. Furthermore, Figure 1 shows a small time shift in routine human behavior. The displayed dots are not just GPS data but a combination of Cell-ID, GPS and Google API. While a few research efforts [24,25] focus on extracting locations from a *combination* of different location data sources (fusion), several efforts focus on collecting and mining location traces from a *single* source of information [26,27] and have demonstrated promising results.

Note that our approach is focused on the data that is being collected from the users’ device (user-centric) and not service provider data, i.e., call detail records (CDR) [28]. Location data can also be obtained from sources including Cell-ID, WiFi, wireless beacons, etc. Cell-ID is too imprecise to be used for location estimation, and, due to limited battery power, users often do not enable GPS. Therefore, a more reliable source, such as a combination of both WiFi and Cell-ID (which are more resource efficient in comparison to GPS alone), should be used for estimating location. In other words, a location estimation algorithm is assumed to extract location from a combination of sensors.

Our change point detection (location estimation) algorithm receives a set of *actions* and *signal type* as inputs and it returns a list of *events*. Actions are a 3-tuple of attribute (sensor name), value (sensor data) and timestamp. Signal type can be WiFi only (e.g., Device Analyzer data) or a combination of sensors. An event includes a location state, start time, end time, and a finite set of actions. The following shows a simplified example of raw sensor data in a time slot, i.e., between 12:00 p.m. to 12:30 p.m. Since the WiFi is not repeated, we consider this time slot as “moving”.

{{name:"call",val:"1800xxx", time:"12:02-12:03"}},

{name:"WiFi",val:"BSSDID_1", time:"12:04"},

{name:"activity",val:"walk-910s.", time:"12:04-12:18"},

{name:"WiFi",val:"BSSDID_x", time:"12:26"}.

The following shows an example of an event with four actions, after change points have been identified and annotated, i.e., “location state”.

{location_state:"moving",time:"12:00-12:30",

actions:{{name:"call",val:"1800xxx",

time:"12:02-12:03"},

{name:"WiFi",val:"BSSDID_1",

time:"12:04"},

{name:"activity",val:"walk-910sec.",

time:"12:04-12:18"},

{name:"WiFi",val:"BSSDID_x",

time:"12:26"} } }.

Note that the algorithm checks the signal type, either Wi-Fi or combination of all location signals. If both Wi-Fi and geographical location exist, the algorithm prioritizes the geographical location over Wi-Fi (due to its superior accuracy).

If it is only Wi-Fi, it searches for consecutive timestamped WiFi logs. If such a sequence exists, and all its elements (i.e., BSSID of WiFi) are unique, this is a sign of a *moving* event. For example, a sequence of not repeated WiFi BSSID as Wx,Wy,Wz is a sign of a moving event. Therefore, a moving event (with its start time and end time) will be created and appended to the *events* list. Otherwise, if it is not a moving event and there is a sequence of elements, but they are not unique (i.e., repeated BSSID), the algorithm identifies them as a *stationary* event. For instance, a 60 min sequence of repeated WiFi BSSID as Wx,Wy,Wa,Wx,Wb,Wy presents a stationary event. If in a time interval of 60 min no WiFi signal exists at all, and all other location signals are not available either, the algorithm creates an *unknown* event. The algorithm uses a time interval of 60 min because it has been identified [1] that the temporal granularity of 60 min has the highest accuracy for routine behavior identification. In other words, this time interval could be assumed as a smoothing factor.

All events include a start time and end time. In short, when the algorithm finds a number of WiFi BSSIDs (let us say names for simplicity) repeated together, it creates a *stationary* event. However, if the WiFi names changes and they are not repeated, it creates a *moving* event. If none of the described cases exist, the algorithm creates an *unknown* event.

If the signal type is not just WiFi and it is a combination of GPS, Cell-ID and a 3rd party location service such as the Google Play service, the algorithm takes a different approach. If geographical coordinates exist (GPS or a similar 3rd party service), the location status is easily computed. To calculate this type of location state, it computes the difference between two consecutive geographical coordinates. If two signals have a distance more than the *distance threshold* $\delta_{d}$ and are equal to or more than the *temporal threshold*$\delta_{t},$ then the algorithm marks the target time frame (event) as *moving*. Otherwise, if the distance is less than $\delta_{d}$ and more than $\delta_{t}$, it marks them as *stationary*. If no location signal appears after $\delta_{t}$ time, then the algorithm creates a new event and marks the event as *unknown*. This event continues until a new location change appears. When a new location signal appears (that creates a different location state), it ends the previous event. Ending an event means the algorithm closes the event with the timestamp of the last element in the dataset. Then, a new event is created with the timestamp of the new location element that has been most recently read.

Note that $\delta_{d}$ is a fixed number and varies between 800 to 1000 m in cell tower installations; e.g., in the city of the UbiqLog experiment, it is fixed to 800 m.

**Figure 3:**
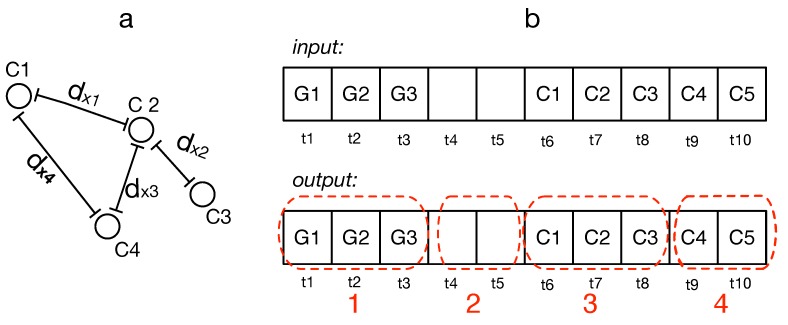
(**a**) Four consecutive locations from Cell-ID; (**b**) four events have been detected, the first three elements contain GPS, and then with two elements marked as unknown. Three later elements, C1, C2, C3 contain cell IDs and show another movement until the point C4. The geographical distance between C4 and both C3 and C2 is less than $\delta_{d}$.

The UbiqLog dataset shows that most of the time GPS is turned off (based on its real-world nature), there are very few GPS logs and they occur mostly when users are navigating. Most location logs are from Cell-ID; and thus it is not possible to precisely estimate location (because of relying on Cell-ID instead of precise coordinate) [25]. In particular, when the location change is noted, there is an ambiguity as to whether the location has truly changed or just the cell tower has changed (i.e., handoff). Nevertheless, there is a fixed precision associated with the location extracted from the Cell-ID. Let us assume the precision distance is $\delta_{d}$, (in the city of the UbiqLog experiment, the precision distance between Cell towers was 800 m) and a temporal precision $\delta_{t}$. To understand this problem, consider the example in Figure 3a. There we have $C_{1},C_{2},C_{3},C_{4}$. If $d_{x1}+d_{x2}>\delta_{d}$, this means that the user is moving. If $d_{x1}+d_{x3}>\delta_{d}$ but the distance between $C_{1}$ and $C_{4}:d_{x4}<\delta_{d}$, then the user is inferred to be stationary and not moving. Therefore, when the algorithm calculates only the distance between two consecutive points, it might face a problem. To resolve this issue, when the location is based on Cell-ID, the algorithm calculates the location distance between *three* consecutive points rather than *two*. Figure 3b shows a trace, which has a combination of GPS(G) and Cell-ID(C) locations. It shows that four Cell-IDs have been recognized and C4 has not been categorized as the same event. Based on cell tower distribution [26], $\delta_{d}$ must be 800 to 1000 m (cell tower distances) to check whether or not there is a location change or not, and five minutes has been assigned to $\delta_{t}$.

Setting $\delta_{t}$ to five minutes is extracted from the evaluation conducted in the data collection experiment [11]. Therefore, in this paper, we do not evaluate the parameter sensitivity of $\delta_{t}$, $\delta_{d}$ and the time intervals.

The computational complexity of the this spatial change point detection algorithm is linear because, even if we assume all locations are Cell-ID, there is a need for a comparison of each element with its two previous ones and thus we require only 3n comparisons (worst case scenario).

### 4.2. Temporal Clustering

The second step is to cluster similar user events of a person based on their *spatial* and *temporal* similarity. Events inside a cluster have the (i) same start time, (ii) same end time and (iii) same location state, which was identified in the previous stage.

**Figure 4:**
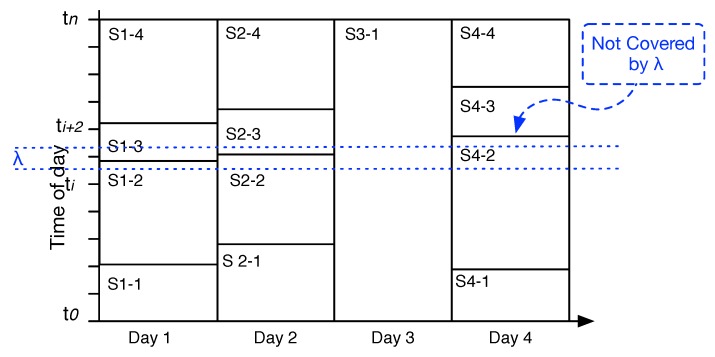
An example of four days with spatio-temporal change points, Day 3 is on a weekend. The fix λ disables the algorithm from recognizing Day 4 events properly in their cluster. In particular, *S1-3*, *S2-3* and *S4-3* should belong to the same cluster. However, by not moving λ, *S4-3* can not fit into the cluster of *S1-3* and *S2-3*.

We interpret similar spatio-temporal events as an indicator for a routine behavior, e.g., commuting to work, going to the park on weekends, etc. Similar events are collected in clusters. As it has been described in Section 2, we need to handle the slope of human timing of similar events and thus use λ. λ can be interpreted as a reasonable “slope interval” to calculate similarities between events. Figure 4 shows a λ interval that covers the start times (lower bound) of two (visually) similar events *S1-3* and *S2-3* from two consecutive days. Clusters are not overlapped.

Algorithm 1 describes our temporal clustering approach. λ and a list of events, inEvents, which are ordered based on timestamps, are inputs. The algorithm iterates through all events; then, it selects the first two days through the initiate method, line 3. $e_{base}$ is the event list of the first day and $e_{second}$ is the event list of the next day. On line 5, similarST method compares the spatial and temporal data of two events from each day. If they are similar, and a cluster with their spatio-temporal properties exists (checked by existsSim) then the algorithm adds both events into their respective cluster, on line 7. Otherwise, if they have similar spatio-temporal properties but no cluster exists with the similar spatio-temporal properties, the algorithm creates a new cluster on line 9 and adds both events into this new cluster. If none of the above conditions are met, both events will be added to tmpNS list (list of orphan events), line 11.

Days are compared sequentially, but there are events that do not occur every day but occur frequently, such as going to the gym twice a week or events originating from weekend activities, e.g., going to the movies. To cluster these events, dissimilar events will go into the tmpNS list (line 11). After the first loop, which compares all events and assigns them to their cluster, the algorithm orders the content of tmpNS based on time, on line 14. Then, it starts iterating through them on line 15. If two consecutive events inside tmpNS are similar, and their spatio-temporal properties are similar to one of the exiting clusters (existSim method on line 15 checks this condition), then these two events will be added to that existing cluster, on line 18. Moreover, these events will be removed from tmpNS because now they have a cluster. If they are not passed to any cluster but their spatio-temporal properties are similar, a new cluster to host them will be created on line 20 and collect them. Nevertheless, if none of these conditions apply, these events do not have any similar events and they will be added to a list of nonsimilar (anomalous) events.

**Algorithm 1:** Temporal clustering of events.

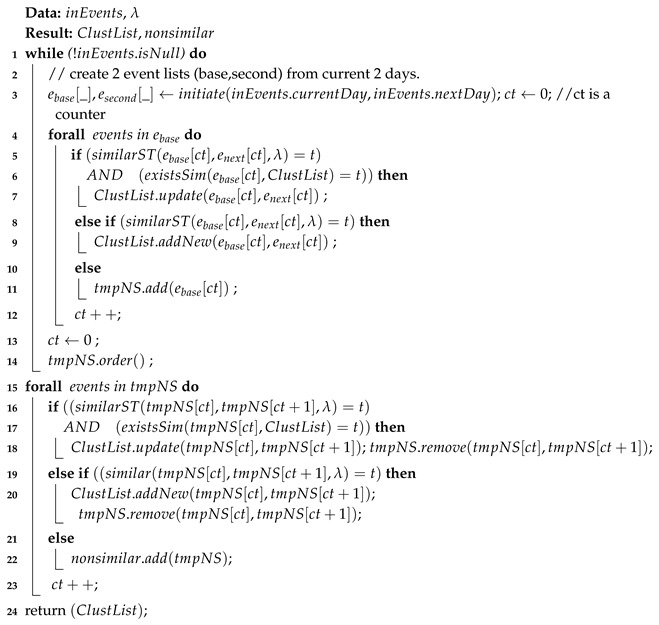



Human behavior slowly evolves over time [1], which means, among other phenomena, similar events and their timings will change over time. To resolve this issue, λ will be moved between days, but it is a fixed variable. In particular, the algorithm will not use one day as a benchmark and then compare the other days to that single day. Figure 4 neglects the spatial property of an event for the sake of readability, and visualizes the problem of not moving λ. The example shows four days within their temporal events identified. *S1-3* and *S2-3* could stay in the same cluster, but *S4-3* is not covered by the λ threshold, despite the fact that we can see it belongs to the same cluster. In addition, *S1-2* and *S2-2* have a similar end time, but if we do not move λ, *S4-2* also lacks a similar end time.

Because of a minor time variety of daily routine behaviors, the λ is changing. If two events are similar, which means their upper bound and lower bound are ≤λ, then λ will be updated as the *average* of upper bound or lower bound between two similar events. Otherwise, λ will be not changed.

Each day will be compared with another day, which requires *n* number of comparisons. In the worst case, the content of orphan event list (nsimilar) is equal to n−1 and again we have about 2(n−1) number of comparisons. This means that the complexity of this algorithm is O(2(n−1)), which is linear.

### 4.3. Detecting Contrasting Events

An individual’s frequent presence in the same location state at the same time does not mean she necessarily engages in exactly the same behavior. In addition, an event may be too prolonged to quantify its content. For instance, a user could stay at home for a day but have significantly different activities, such as recuperating from an illness or working from home. To identify such differences, we propose a novel contrast behavior (CB) detection approach for events *inside* a cluster. Our CB detection algorithm is inspired by contrast-set mining algorithms [13]. Some research considers contrast-set mining as a rule discovery [29], but we have a different interpretation, tailored for mobile data that are multivariate temporal data.

Algorithm 2 presents a method to compare the actions of each event *inside* a cluster. The algorithm receives a cluster, inC, and ω. As previously described, ω is the threshold for uncommon actions in each event. The algorithm identifies the contrasting events in each cluster and at the end reports for each cluster how many of its members (events) are contrasting and how many of them are similar. The result of this algorithm is useful for searching because it enables the search algorithms to prioritize the clusters, based on the number of similar events.

**Algorithm 2:** Contrast behavior identification from events inside a cluster.

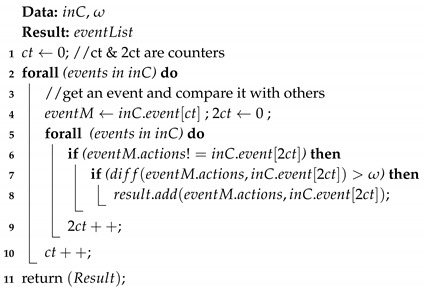



On line 2, the algorithm iterates through the number of events in a cluster (line 5) and compares each event (eventM) actions with other events inside that cluster, on line 6. The diff method (line 7), compares two events and, if the number of different actions is larger than the ω threshold, then those events are counted as contrasting behaviors. This comparison is measuring the *exact* similarity between each action. We did not use other similarity metrics such as Jaccard coefficient because our empirical experiments show that the number of actions of events inside the cluster are either equal or the difference is very insignificant. At the end, they are collected in the Result set and returned. This comparison is measuring the *exact* similarity between each action.

A large number of dissimilar events indicates that the user’s activities are not routine. The ω value is application dependent. It also depends on the temporal event size, the purpose to which outputs are used, and how that benefits from our approach. For instance, if an event size is about a day (e.g., a device is stationary during the day) contrasting behaviors do not reveal much about the underlying semantics of the data. Assuming *n* number of events are inside a cluster, each event inside a cluster is compared with other events in the cluster. Therefore, the algorithm has $n^{2}$ comparisons and its complexity will be $O(n^{2})$. However, the number of comparisons is limited to only the number of events inside a cluster. Therefore, the number of comparisons is small (e.g., two to eight in a UbiqLog dataset) and thus the performance overhead is insignificant. Section 5.3 reports this cost in detail.

Note that the contrast behavior detection provides a minor semantic improvement, i.e., annotation, on the actions inside a cluster and still more knowledge extraction is required on the data. In particular, contrast behaviors will be used mainly to order clusters for the search. The implementation of the annotation, such as geo-fencing, drives the conversion of sensor data to a higher level of information in the task of the application that uses our algorithms. Therefore, there is still a need for manual annotation, but our approach significantly reduces it. For instance, for the ground truth dataset, we have implemented a simple annotation, based on users’ manual labels, e.g., home, gym, work, etc. Users annotate one event only once in a cluster, and then it will be distributed among other events in that cluster.

## 5. Experimental Evaluation

This section demonstrates the utility and efficiency of the proposed algorithms in detail. In addition, we report about the spatio-temporal clustering impact on search execution time reduction and battery utilization, which is our main objective. Firstly, we begin by evaluating the event detection. Then, we demonstrate our experiment for cluster detection, its impact on search time and energy use. Afterwards, we demonstrate the contrast behavior detection accuracy and its impact on searching.

### 5.1. Event Detection

To evaluate the efficiency of the event detection algorithm, first we have built a ground truth dataset. This dataset will help us to analyze the accuracy of detected events and finding the optimal value for λ.

#### 5.1.1. Ground Truth Dataset

We have created a ground truth dataset that includes manual labels. In particular, 10 participants have used UbiqLog, and 10 other participants have used a Device Analyzer for two weeks. Participants included 7 females and 13 males, (mean age = 27.3, SD = 4.5). Participants have manually labeled their location state changes. They label only one event in each cluster, i.e., first event, and the rest of the labels will be distributed automatically to the other events in each cluster. Participants can choose between one of these predefined labels [30]: “Commute”, “Sport”, “Home”, “Work”, “Leisure” and “Other”. They have installed a simple tool that enables them to choose their location state changes manually from the given list.

The resulting dataset, with λ=30’, contains 34 distinct clusters (not shared between users). Our algorithms have extracted 237 events, 69 of them were contrasting events, ω=2. Later in the evaluation section, we describe the policy of choosing ω=2.

#### 5.1.2. Accuracy of Detected Events

**Figure 5:**
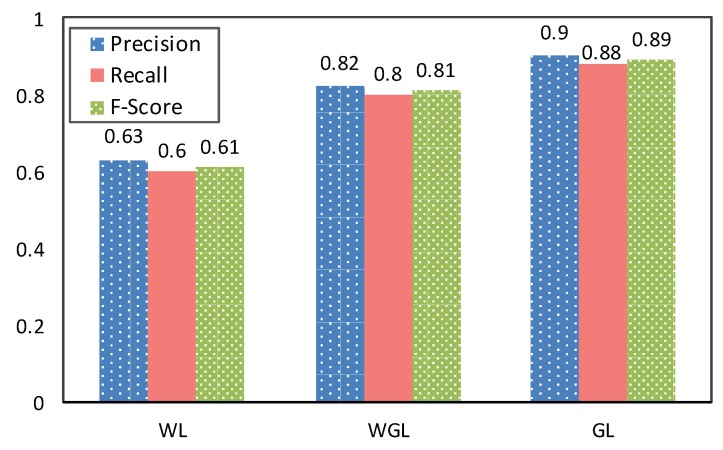
Accuracy of the three different location state estimation approaches based on available data type(s); Wifi Location (WL), Wifi/Geographic Location (WGL) and Geographic Location (GL).

The first question for the event detection evaluation is whether the identified location state of the event is correct. Based on real-world settings, the location state could be detected from three different states: (i) there are no geographical coordinates, and only WiFi data can be used, i.e., WiFi Location (WL); (ii) WiFi combined with geographical coordinates, i.e., WiFi/Geographical Location (WGL); (iii) the GPS sensors on the phone are always on, and location is stored using only geographical coordinates, i.e., Geographic Location (GL). It is unrealistic to assume uninterrupted 24/7 sensing of geographical coordinates. Nevertheless, participants were asked to implement all three statuses during the experiment and never turn off their phone or use airplane mode. Their labels have been used to calculate “precision”, “recall” and “F-score” for the evaluation. In particular, “true positives” are location states that are similar both in user labels and the system, and they are not “unknown”. “False positives” are location states that are identified by the system and not “unknown”, but users have labeled them differently. “False negatives” are location states that have been identified by the system as “unknown” and the users have labeled them either as “moving” or “steady”. Clearly, they did not use the “unknown” as a label. Figure 5 reports about the accuracy of these three described states. The low number of false positives leads precision to be higher than recall in WGL and GL. Due to several false negatives, WL has a lower recall than other methods, which is due to the WiFi sensor that is mostly turned off to preserve the smartphone battery. In other words, in the absence of WiFi and GPS, the system marks these data as “unknown”, but clearly participants have provided labels.

Furthermore, Table 3 reports the accuracy of location states based on the type of location state (stationary vs. moving). In other words, Figure 5 presents the accuracy of different sensor settings, whilst Table 3 presents how accurate each sensor setting can measure the location state (moving vs. stationary). It shows that using GPS significantly increases the accuracy of detecting moving events. As expected, WiFi alone (WL) has the lowest accuracy because of the lack of geographical coordinates. Note that, since the number of movement and steady (stationary) events are not always equal, we can not average results of Table 3 to get the result of Figure 5. Therefore, we have asked participants to label each event individually.

### 5.2. Clustering

To evaluate the utility of the proposed clustering algorithm, first we compare scalability with other clustering methods along with a comparison of quality. To compare our algorithm with representative algorithms, we have converted the combination of sensor name and its value to the number, and we have normalized time of day with five minutes precision (temporal granularity [1]) and converted it to a number.

Through parameter sensitivity analysis, we get the most accurate results with the following parameters for clustering algorithms: K = 9 for k-mean, minPts = 3, eps = 5 for DBSCAN and k = 8 for the hierarchical clustering.

Moreover, to demonstrate the capability of the clustering algorithm to handle non-daily routines, we analyze the accuracy of our clustering algorithm in two different modes (neglecting orphan events versus using them). Then, we report about the parameter sensitivity of λ. Afterwards, we demonstrate the significant clustering impact on search.

#### 5.2.1. Scalability of Clustering Algorithm

The execution time and maximum memory usage are indicators of the scalability of an algorithm. Here, we compare our clustering execution time and memory usage to well-known clustering algorithms, i.e., K-means, Hierarchical clustering (HCA) and DBSCAN. We have chosen these algorithms because most existing works on mining mobile data were focused on using one of these representative algorithms.

Note that there is no state-of-the-art spatio-temporal clustering developed for smartphone data, i.e., to extract location from WiFi and geographical coordinates. Therefore, we have chosen to compare our clustering approach with well-known methods.

Since the algorithm should run on small devices, this experiment ran on a smartphone using an SPMF library [31] to implement the clustering algorithm. The test device was a Moto G 2nd Gen. (Motorola, Chicago, IL, USA) with a quad-core 1.2 GHz CPU and 1 GB RAM. Our clustering algorithm is abbreviated as ST ( Spatio-Temporal) in Table 4. This table summarizes the execution time and the maximum memory used for each clustering algorithm. Table 4 reports the average numbers from both datasets. The λ were set to its optimal value, i.e., 30 min, which will be analyzed later.

Results in Table 4 show significant improvements over other algorithms in both maximum memory use and execution time, for both datasets.

#### 5.2.2. Quality of Clustering Results

In order to measure the quality of our ST clustering algorithm, we have used the Dunn Index (DI) [32], entropy (EN) [33] and distance comparison (WB). To calculate the WB, we have divided the average distance *within* the clusters to the average distance *between* the clusters. Table 5 reports data for all users in both datasets.

Table 5 shows that our clustering algorithm (ST) outperforms others in WB and EN, on both datasets. Only the DI, with the HCA algorithm in the UbiqLog dataset, returns a higher value (better) than ST. As previously stated, our clustering algorithm handles nondaily routines as weekend behaviors. Table 6 compares the quality of our clustering algorithm in two modes (i.e., ST1 and ST2). ST1 neglects the orphan events analysis in the clustering algorithm. ST2 takes into account orphan events and thus its accuracy should be higher. Table 6 shows the higher accuracy in ST2 compared to ST1 and thus demonstrates the ability of our algorithm to correctly identifying non-daily behaviors. In particular, at the first iteration of clustering 33% of events were orphan events, which do not stay in a cluster. During the next iteration, only 11% of events remained as anomalous events, and 22% were assigned to existing clusters. Calculating orphan events does not have any impact on quantitative results, including execution time and battery utilization. Its only impact is on the quality of clustering.

Analysis in Table 4 and Table 5 has been conducted on all 70 users. However, results of Table 6 require subjective annotation. Therefore, we have conducted this experiment on 20 ground truth users and not on all users.

#### 5.2.3. Parameter Sensitivity of Lambda

**Figure 6:**
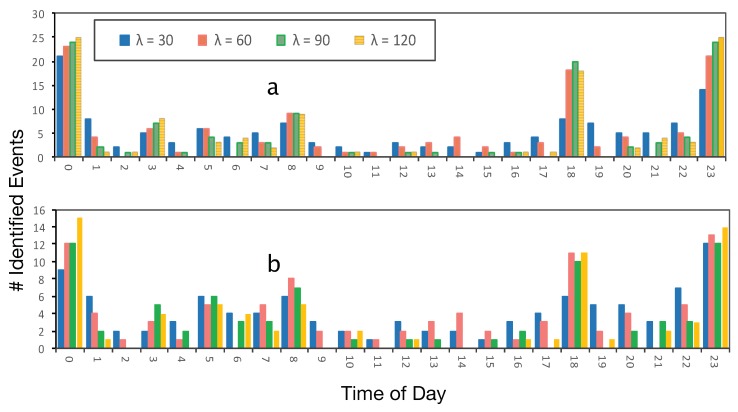
Impact of different λ values on the number of events detected during the day. (**a**) is the UbiqLog dataset and (**b**) is the Device Analyzer (best viewed in color).

λ is the configurable parameter that has been used as a boundary for identifying similar events and clustering them together. In order to analyze the sensitivity of λ, we report on four different values for λ: 15, 30, 60 and 90 min. Figure 6 reports about the number of events that have been identified in each dataset, based on different λ values and time of the day (from 12:00 a.m. to 11:59 p.m.). This figure reports on all users as well.

In particular, increasing λ results in a fewer number of clusters because of short time events, which are neglected (less than 60${}^{\prime }$ or 90${}^{\prime }$). However, at some specific times, a larger λ can identify more events, which appears as spikes in Figure 6. For instance, λ with large values, i.e., 60${}^{\prime }$, 90${}^{\prime }$ and 120${}^{\prime }$, can identify more events near bed time and commuting times. There are three main spikes in both datasets including leaving for work/school, arriving home and near bed time. The first spike, which is bedtime around 00:00${}^{\prime }$, is connected to the last spike, which started around 11:00 p.m. Therefore, we can observe three major spikes and not four spikes.

Based on Figure 6, different values of λ (except 15 min) do not have significant differences on the number of events. Setting lambda to 15 min leads to a fewer number of events in a cluster. Other λ have approximately similar results. On the other hand, the ground truth dataset users have evaluated the precision of different λ settings. Table 7 reports the WGL settings with different λ. In particular, 30 and then 60 min have the highest accuracy, followed by 90 and 15 min. Therefore, based on the identified accuracy in Table 7, we identify that the optimal value of lambda is 30 min, followed by 60 min. Note that all routine events are not associated with 30’ temporal differences. For instance, calling a friend every day is not precise to fit into a 30’ slope. Nevertheless, there are more precise events, such as arriving at work, which neutralizes the impact of those imprecise routine events. λ is a parameter that reports a single best estimator for all behaviors.

Results in Figure 6 were based on using all users’ data. However, results in Table 7 are from 20 ground truth users because it requires manual labeling to be able to identify the accuracy.

#### 5.2.4. Search and Battery Impact

The cluster-based search mechanism can be understood as a one-dimensional ‘index’ that has been used to accelerate searching. Clustered data are amenable to further indexing (e.g., bitmap, B-trees, etc.), but here we confine our attention just to the improvements obtained by our re-representation of the data. As previously noted, the problem of searching sensor data has not been widely explored for mobile and wearable devices. Mobile/wearable sensing applications can collect large amounts of data; however, searching them is a resource intensive process and thus a simple brute force search on raw data is not feasible.

Since all 70 users can not annotate their data, we only use our ground truth users. Participants of the ground truth dataset manually segmented their daily events with the following words: “Commute”, “Home”, “Work”, “Leisure” and “Other”. We have implemented a Wi-Fi and location geo-fencing component to automatically replicate labels to the other events in the same cluster. Then, we assign the described labels to all 70 users (based on labels’ distribution in the time of day) by using our ground truth labels as a template, e.g., morning events are usually ‘commute’ or ‘work’, evening events are ‘Home’ or ‘Leisure’. Label assignments are not necessarily correct, but the objective here is to prepare them for searching by disregarding their semantics.

**Figure 7:**
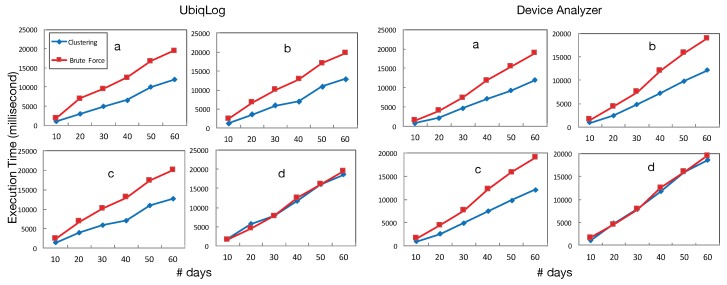
Four different search execution time samples for UbiqLog and Device Analyzer. The *y*-axis shows execution time in milliseconds and the *x*-axis shows the number of days that will be searched.

To test searching on the labeled data, we have considered two search algorithms. One is brute force, used as a baseline, which has been compared to our clustering algorithm. These experiments have used the same device with annotated location data. Figure 7 shows two samples from the Device Analyzer dataset and the UbiqLog dataset. We have considered four types of search for each user sample, which includes both time and location (due to spatio-temporality of clusters):

(i) search with time (T), location state (L), sensor name (S) and sensor data (D) (Figure 7a, e.g., *How long on average do I spend playing games, while at home, after 9:00 p.m.?*
(ii) search with L, S, D, Figure 7b, e.g., *How many SMS do I receive, on average, while at work?*
(iii) search with T, S and D Figure 7c, e.g., *When was the last time I went running?*
(iv) search with S, D, Figure 7d, e.g., *How often did I call my parents?*

To parse the user input, we have used a light query engine [34] that can parse Quantified-Self queries on mobile and wearable devices. Numbers presented in Figure 7 are averaged among all users in each dataset, i.e., they present an average user time required for queries on the smartphone. However, Figure 7d has no notion of time or location. Figure 7 demonstrates the significant impact of the clustering algorithm on search execution time for both datasets. The improvement increases with the size of the data. However, query (d) that does not include either a notion of time or location does not have any significant difference with the brute force method.

Note that there is no space overhead, since the clustering merely changes the storage file structure. The only overhead is the time required for the cluster calculation, which can be done at off-peak times, e.g., when the device is charging. Moreover, the improvements in speed do not come at a cost of accuracy; the search of our condensed data is *admissible*, producing *identical* results to the search over the original raw data.

As noted previously, battery utilization is a major challenge in small devices [35]. Here, we also demonstrate the battery utilization differences between our cluster-based search versus the brute force search (baseline). Table 8 shows the impact of our cluster based search on battery efficiency, as measured in microWatts (mW) and significant improvements over brute force search. This table reports the averaged battery utilization among all four types of aforementioned queries.

### 5.3. Contrast Behaviors

Three experiments have been used to evaluate our Contrast Behavior (CB) detection approach. First, the parameter sensitivity of ω and its impact on the quantity of CBs was analyzed. Second, characteristics of CBs were determined by analyzing the correlation between time of the day and event duration. The third experiment examined the search execution time based on prioritizing clusters ordered by their number of CBs.

#### 5.3.1. Parameter Sensitivity of Omega

It is notable that the Device Analyzer dataset contains only hardware configuration or changes in the hardware properties, thus its data objects are not necessarily correlated with human behaviors. Therefore, the second evaluation used the UbiqLog dataset (35 users). ω is a configurable parameter we have introduced to be used for CB identification. Similar to that for other clustering algorithms, such as K-means, there can not be an optimal value for ω. This variable is open to settings by developers using this algorithm. For instance, a user might frequently go to a coffee shop in the evenings (similar spatio-temporal event, assigned to the same cluster) either for work, or chatting with friends. Both scenarios take place in the coffee shop at about the same time, but her other actions could be different. If the goal of the target application is to detect only appearances in the coffee shop, and not other actions, ω could be set to zero. However, if the goal of the target application is to detect reasons for being in a coffee shop, ω should be set to more than zero, to detect dissimilar actions. As another example, a user either goes bird watching or golfing to a golf course. These activities could be identified via a comparison between wrist movement data, in which case ω can be equal to one.

**Figure 8:**
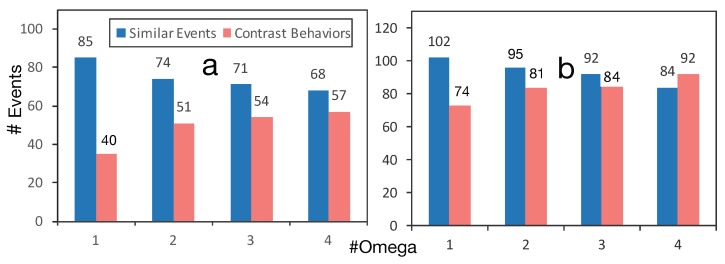
Parameter sensitivity of ω in (**a**) UbiqLog dataset and (**b**) Device Analyzer dataset.

To gain a deeper understanding about the events inside each cluster, we have tested five different variables for ω: 1,2,3,4 and 5. Figure 8a reports about the number of detected events among all users in the UbiqLog dataset and Figure 8b reports on the Device Analyzer dataset. As it has been shown, increasing the value of ω decreases the number of similar events, thus resulting in more events from each cluster being considered as CBs. Figure 8 shows that the boundary for setting ω is different between two datasets. For instance, setting ω to three and larger creates more CBs than similar events, in the Device Analyzer dataset. Nevertheless, ω is not dataset dependent, increasing it simply reduces the chance of having more similar entities and vice versa.

#### 5.3.2. Characteristics of Contrasting Events

**Figure 9:**
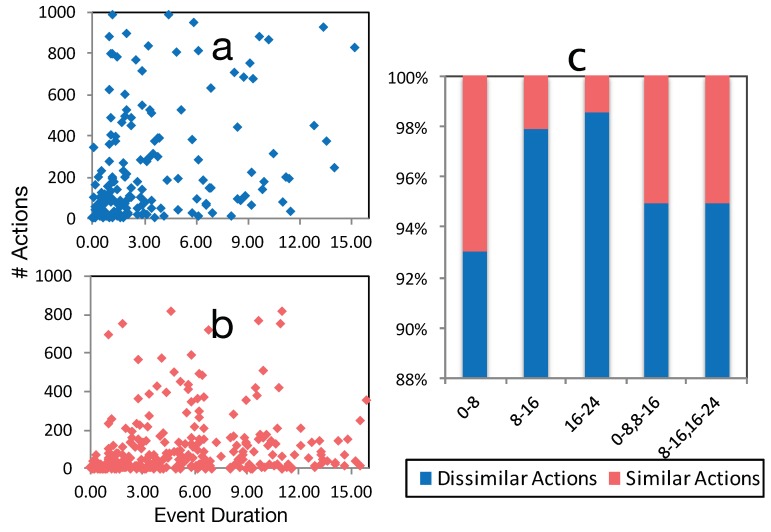
(**a**) distribution of similar actions (not events); (**b**) distribution of dissimilar actions; both (**a**,**b**) were based on event duration using ω=3; (**c**) ratio of similar actions inside events of each cluster, distributed among different temporal segments.

Our CB identification algorithm is capable of identifying dissimilar actions for routine behaviors. We begin with the observation that for most people, their range of behaviors is very limited between 12:00 a.m.–8:00 a.m. (while sleeping), more varied between 8:00 a.m.–4:00 p.m. (during work or school) and highly varied after 4:00 p.m. (during leisure time). To show this, we use an approach similar to [36] and segment days into three temporal divisions: 12:00 a.m.–8:00 a.m., 8:00 a.m.–4:00 p.m. and 4:00 p.m.–11:59 p.m. We consider only events shorter than 16 h. Figure 9b which is done for ω = 3, shows the average distribution of the ratio between similar and dissimilar actions belonging to events from a single cluster. Morning events have the fewest number of CBs, perhaps because behaviors following sleep tends to be routine. In contrast, evening events have a higher number of CBs, supporting our initial hypothesis.

In order to evaluate these findings, we have created a contingency table of temporal segments (8 h and 16 h segments) and dissimilar and similar actions. Similar to [13], we use the chi-square test to statistically evaluate our interpretation. The result (*p* < 0.05) supports our assumptions about the distribution of contrasting behaviors among different temporal segments.

Another interesting finding (Figure 9a) shows that the distribution of dissimilar actions is highly concentrated on short temporal events. In contrast, events longer than three hours have far fewer dissimilar actions. This maps to our intuition that routine spatio-temporal behaviors are longer by their nature, e.g., staying 9 h at work, sleeping 7 h per day, etc. To validate this observation, we have calculated the odds ratio of the number of similar actions versus dissimilar actions, and the number of actions, which have less than three versus more than three hours duration. The result of the odds ratio calculation indicates clusters that last less than three hours are 8.2 times more likely to have contrasting behaviors than clusters with events longer than three hours.

#### 5.3.3. Contrast Behavior Impact on Search

Understanding the characteristics of contrasting behaviors could improve the search execution time by prioritizing clusters, i.e., first, the system searches clusters with a lower number of CBs and then searches clusters with a higher number of CBs. In order to evaluate this hypothesis, we have ordered the clusters based on the number of their CB ratio to similar events, i.e., a cluster with a higher number of CBs gets a lower rank. There is a small cost of ordering clusters, but, due to the small number of clusters, it is insignificant. In Section 5.2.4, we execute two different search commands for each of the described search conditions. The first search command gets the data from the cluster with the largest number of CBs, and the second command, in contrast to the first one, gets the data from the cluster with the highest number of similar events.

**Figure 10:**
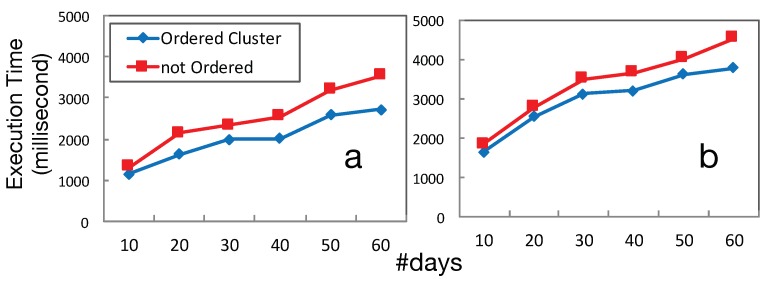
Improvement of search execution time (in milliseconds) by ranking clusters based on their number of contrast behaviors. (**a**) UbiqLog and (**b**) Device Analyzer dataset.

Figure 10 shows the differences between searching clusters ordered by number of CBs versus not ordered. As it has been shown, there is a slight improvement in the search execution time, especially when the number of days increases. This result is in line with our initial hypothesis, and thus ordering clusters based on their CBs can reduce the search execution time.

## 6. Related Work

Based on our contributions reported in this paper, we categorize related works into three different categories. First, we review works that focus on spatio-temporal segmentation or clustering from mobile devices and their sensors, i.e., WiFi, GPS and Cell ID. Then, we review works that focus on detecting patterns of location changes or location of interests. Afterward, we review works that try to extract events from daily life events. These works either rely on sensor data or are user-centric and focus on daily activities of users. There are several works that attempt to estimate users’ behavior from cell tower data, but since our approach is focused on data from users‘ devices, we do not list them here. For example, Ghahramani et al. [37] focuses on identifying geographical hotspots based on smartphone concentrations, by analyzing spatial information of cell IDs collected from a telecom provider. In addition, there are some works that employ other mediums for location information mining, such as social media [38], which we do not list them here.

### 6.1. Spatio-Temporal Segmentation

Zhou et al. [26] provide one of the earliest works in spatio-temporal clustering of daily location changes. To address sparse and noisy GPS data, they provide a density-based algorithm for clustering because density based algorithms can remove noise in the final clustering results. There are several works that have focused on geographical location. For instance, Mokbel et al. [39] propose a three-phase algorithm (hashing, invalidating and joining) to parse continuous spatio-temporal queries. Zhang et al. [40] use text as a raw material for location with an index structure that reduces the search space using spatial and keyword base pruning. A recent example is introduced by Christensen et al. [41] which focuses on facilitating accessing spatio-temporal through interactive spatial online sampling techniques. There are other indexing methods that operate on multi-metric characteristics of data [42], such as location or time. However, our work uses spatio-temporal similarity of data for indexing.

### 6.2. Location and Spatial Information Mining

There are several examples of research that benefit from smartphone location logs, i.e., GPS, WiFi, Cell ID, to identify locations of interest and daily movement patterns. Reality mining [16], is one of the first efforts toward identifying behavior from smartphone sensor data and so created a benchmark dataset that is still in use, e.g. [14]. For instance, Farrahi et al. [14] use distant n-gram topic modeling to mine latent location data and avoid parameter dimension explosion. Recently, the uncertainty of a realistic deployment has been taken into account and there are some works that try to support uncertainty while mining for location data originating from unreliable smartphone sensors too [43]. Placer [44] is another work that labels location data in geographical coordinates based on individual demographics, the timing of visits, and nearby businesses. Our approach uses *a combination of sensors* rather than just location as a unit of human behavior. Furthermore, our approach does not focus on location itself and relies on the movement of the user. Ma et al. [36] describe a normalization algorithm that transforms low level geographical coordinates to location terms, i.e., “work” or “home”, based on time of day. Their results are promising, but our generalization of location is more detailed than the work/home approach. ePeriodicity [45] is another more recent algorithm that uses probabilistic measures to identify temporal periodic behavior from GPS data. It can tolerate sparsity and noise, but it is limited only to GPS data. Note that our work is not about trajectory partitioning. Our approach is focused on user behavior and the events are mutually exclusive, and independent from the trajectory of movement.

### 6.3. Daily Event Detection

There are promising works that try to understand daily life events from continuously collecting images. Doherty et al. [46] have provided one of the initial works in this area with their event segmentation of digital photographs. Gomi and Itoh [47] propose a method to categorize and annotate personal photograph collections based on location, time and individuals appearing on the photos. Kelly and Jones [48] describe the problem of searching large personal archives and use Galvanic Skin Response (GSR), a marker of emotional arousal, to index life events. In particular, a fluctuation of GSR, with temporal correlation to images, highlights related life events in the life log dataset. There are context sensing approaches toward data collection from multiple sensors and to identify human behavior such as JigSaw [49] or Lasagna [50]. Lasagna enables searching physical activities. It extracts common bases of motion data by analyzing similarities between raw sensor data, and, by using deep neural networks, it identifies and annotate activities. Separate from images, there is another group of works that tries to detect holistic daily patterns from raw mobile data [30,51,52]. For instance, Lifestream [52] proposes general statistical change point detection based on the distribution of behavior. Our work focuses on annotating raw data based on their spatio-temporal properties and pattern detection using spatio-temporal annotation as the building blocks of clusters.

## 7. Conclusions and Future Work

In this work, we propose a pipeline of three algorithms specifically designed to extract spatio-temporal knowledge from mobile data. The algorithms exploit the spatio-temporality of human behavior to identify and cluster daily spatial events based on their temporal similarities. We also suggest a contrast behavior detection algorithm that offers semantic enrichment of mobile data.

Spatio-temporal annotation, demonstrated on two large real-world datasets: (i) significantly improves search execution time, especially when searching a large number of days; (ii) improves battery efficiency while searching for data two real-world context sensing smartphone datasets. The clustering and contrast behavior detection algorithms each have only one parameter to configure and we report about the optimal parameter for each algorithm as well.

Future work will attempt to quantify the temporal variability of human behavior over longer periods (to model concept drift) and integrate this clustering facility into an end-user application. Then, we will study user experience while using such a personal data searching facility.

## Figures and Tables

**Table 1:** Number and types of sensors in the UbiqLog dataset.

Sensor Name	Num. of Instances
WiFi	8,750,111
Location	725,560
SMS	28,849
Call	99,022
App. Usage	45,803
Bluetooth	117,236
Activity State	15,641
All Data	9,782,222

**Table 2:** Numbers and types of sensors for 35 random users’ data from Device Analyzer dataset.

Sensor Name	Num. of Instances
WiFi	2,288,642
Application	98,392,622
Phone	15,719,384
SMS	104,643
Bluetooth	9620
Analytics	2910
Power	5,716,330
System	1,051,175
Audio	4,839,668
CPU	1,143,736
Image	2,281,293
Video	152,397
Memorycard	83,572
Net	232,954
HF	16,687
All Data	132,035,633

**Table 3:** Average accuracy of different sensor settings for each location state.

	WL		GL		WGL	
	Moving	Steady	Moving	Steady	Moving	Steady
F-score	0.26	0.91	0.90	0.78	0.90	**0.92**
Precision	0.48	0.88	0.85	0.74	0.93	**0.94**
Recall	0.11	0.93	**0.96**	0.79	0.92	0.92

**Table 4:** Execution time (in seconds) and maximum used memory (in MB) comparison between our clustering algorithm ( ST) and other algorithms.

Algorithm	UbiqLog		Device Analyzer	
	Exec. Time	Memory	Exec. Time	Memory
HCA	206.7	135.81	314.3	297.18
DBSCAN	39.51	34.77	45.21	38.50
K-means	56.83	36.48	59.84	41.06
ST	**27.19**	**34.24**	**39.37**	**38.23**

**Table 5:** Quality comparison between ST and representative clustering methods in two datasets.

Algorithm	UbiqLog			Device Analyzer		
	DI	EN	WB	DI	EN	WB
HCA	**0.0124**	1.342	0.626	0.0092	1.146	0.482
DBSCAN	0.0070	2.827	0.187	0.0052	2.931	0.153
K-means	0.0085	2.149	0.174	0.0065	3.149	0.144
ST	0.0103	**1.284**	**0.742**	**0.0120**	**1.137**	**0.592**

**Table 6:** Comparision of the clustering approach by analyzing orphan events (ST1) and not analyzing them (ST2).

Clustering Algorithm	Precision	Recall	F-Measure
ST1	0.78	0.72	0.75
ST2	**0.81**	**0.74**	**0.78**

**Table 7:** Accuracy of different values for λ.

	Lambda Values			
	15${}^{\prime }$	30${}^{\prime }$	60${}^{\prime }$	90${}^{\prime }$
F-Score	0.77	**0.91**	0.85	0.78
Precision	0.77	**0.90**	0.87	0.83
Recall	0.78	**0.92**	0.85	0.82

**Table 8:** Energy use in micro-Watt (mW) comparing brute force and our cluster based search operations.

# Days	Device Analyzer		UbiqLog	
	Clustering	BruteForce	Clustering	BruteForce
10	**183**	354	**098**	257
20	**280**	401	**120**	285
30	**284**	417	**145**	332
40	**325**	446	**196**	374
50	**357**	507	**217**	398
60	**401**	580	**279**	404
